# The effects of sex-biased gene expression and X-linkage on rates of adaptive protein sequence evolution in *Drosophila*

**DOI:** 10.1098/rsbl.2015.0117

**Published:** 2015-04

**Authors:** Victoria Ávila, José L. Campos, Brian Charlesworth

**Affiliations:** Institute of Evolutionary Biology, School of Biological Sciences, University of Edinburgh, Edinburgh EH9 3FL, UK

**Keywords:** Faster-X effect, sex-biased gene expression, *Drosophila*, sexual selection, recombination

## Abstract

A faster rate of adaptive evolution of X-linked genes compared with autosomal genes may be caused by the fixation of new recessive or partially recessive advantageous mutations (the Faster-X effect). This effect is expected to be largest for mutations that affect only male fitness and absent for mutations that affect only female fitness. We tested these predictions in *Drosophila melanogaster* by using genes with different levels of sex-biased expression and by estimating the extent of adaptive evolution of non-synonymous mutations from polymorphism and divergence data. We detected both a Faster-X effect and an effect of male-biased gene expression. There was no evidence for a strong association between the two effects—modest levels of male-biased gene expression increased the rate of adaptive evolution on both the autosomes and the X chromosome, but a Faster-X effect occurred for both unbiased genes and female-biased genes. The rate of genetic recombination did not influence the magnitude of the Faster-X effect, ruling out the possibility that it reflects less Hill–Robertson interference for X-linked genes.

## 1. Introduction

The differences between the modes of inheritance of genes on the X chromosome and the autosomes are expected to affect their patterns of variation and evolution [1]. In particular, there may be a ‘Faster-X’ effect. With male heterogamety, rare variants at loci on the hemizygous X chromosome are exposed to natural selection in males even if they have recessive effects on fitness, whereas recessive autosomal variants in randomly mating populations are carried mainly in heterozygotes and largely escape selection. If adaptive evolution is mostly caused by the fixation of new mutations, there may thus be a faster rate of substitution of beneficial X-linked mutations compared with autosomal mutations [1]. Such a difference in the rate of evolution is not expected for mutations with female-limited fitness effects and is most likely to occur for mutations with male-limited fitness effects. When adaptive evolution uses standing variation rather than new mutations, a Faster-X effect is less likely [1,2].

These theoretical results have stimulated several empirical investigations, using data on rates of molecular evolution in several different groups of organisms to test for Faster-X effects. These studies have mostly involved the use of statistics such as *K_A_*/*K_S_*, which measures sequence divergence for non-synonymous substitutions relative to synonymous or silent substitutions; overall, the results have been somewhat mixed [1]. Whole-genome sequence information on *Drosophila melanogaster* and its relatives has shown, however, that *K_A_*/*K_S_* is higher for the X than the autosomes [3,4]. Comparisons of polymorphism levels for non-synonymous and synonymous variants, together with non-synonymous and synonymous divergence among species, suggest that this pattern reflects a higher rate of fixation of selectively favoured non-synonymous mutations on the X chromosome compared with the autosomes [5]. In addition, male-biased gene expression has often been found to be associated with a faster rate of evolution of protein sequences [6], and there is an indication that the Faster-X effect in *D. melanogaster* is associated with male-biased gene expression [7].

Here, we describe results obtained with the DFE-alpha method of Eyre-Walker & Keightley [8] for estimating the extent of adaptive evolution of protein sequences, using a polymorphism dataset on *D. melanogaster* that we have previously analysed [5]. By partitioning genes according to their levels of sex-biased gene expression, we confirm the existence of a Faster-X effect on adaptive evolution, as well as an effect of male-biased gene expression, but find no evidence for a strong association between Faster-X effects and sex-biased gene expression.

## 2. Material and methods

A whole-genome, next-generation DNA sequence polymorphism dataset of 17 haploid genomes from a Rwandan population of *D. melanogaster* was obtained from the *Drosophila* Population Genomics Project [9] and analysed for a set of genes chosen with the criteria described in [5]. We used *D. yakuba* as an outgroup; details of the criteria used to obtain coding sequences that are orthologous between *D. melanogaster* and *D. yakuba* are described in [5]. Female recombination rates for these genes, in terms of centiMorgans per megabase, were obtained from [10]. These rates were multiplied by two-thirds for X-linked genes and by one-half for autosomal genes, respectively. This procedure provides estimates of ‘effective recombination rates’, and takes into account the absence of recombination between homologous chromosomes in males, and the fact that X-linked genes and autosomal genes spend two-thirds and one-half of their time in females, respectively [5]. This allows comparisons between X-linked and autosomal genes that experience the same rates of recombination as far as evolutionary processes are concerned [5].

The ratios of male to female expression levels in whole adult flies were obtained for each gene from the Sebida expression database v. 3.0 (www.sebida.de) [11], and genes were classified as male, female or unbiased on the basis of exceeding the threshold for a 20% false-positive detection rate for a sex difference in expression level (http://141.61.102.17/sebida/content/references/statisticalanalysis.html). Use of this approach means that our analyses are comparable with those of Baines *et al*. [7], although the previous study included less expression and population genetic data.

The different categories of genes described below were analysed using the DFE-alpha method of Eyre-Walker & Keightley [8], following Campos *et al*. [5]. This uses polymorphism and divergence data to estimate the proportion of non-synonymous differences between a species pair that have been fixed by positive selection (*α*), and the ratio of the rate of substitution of positively selected non-synonymous mutations to the rate of synonymous substitutions (*ω_a_*). Genes were classified according to sex differences in gene expression and recombination rate in two ways, keeping X-linked (X) and autosomal genes (A) separate. The first method was to group them into low-, medium- and high-recombination rates, according to the classification of Campos *et al*. [12]; genes in regions that lack crossing over according to the criteria in [5] were excluded from our analyses. These groups were then divided into male-biased, unbiased and female-biased categories. The second method was to divide genes into groups of approximately 80, according to their recombination rates and class of sex-biased gene expression. Further details are given in the electronic supplementary material, tables S1–S7.

## 3. Results

**Figure 1.**
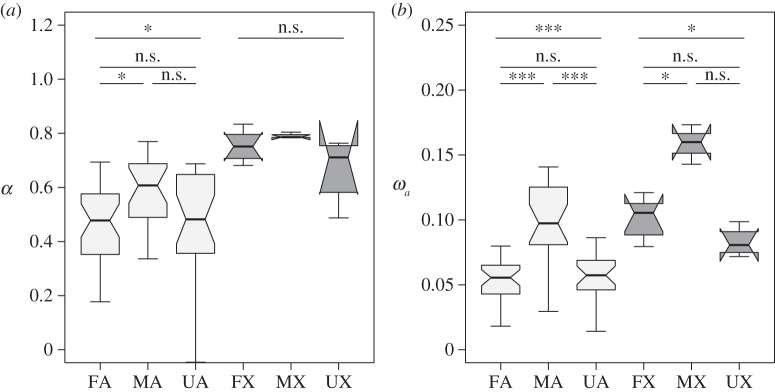
Boxplots of (*a*) *α* and (*b*) *ω_a_* for female-biased (F), male-biased (M) and unbiased (U) genes on the autosomes (A) and on the X chromosome (X). * and *** denote *p* values of 0.05 and 0.001, respectively, from Mann–Whitney *U*-tests; n.s. denotes lack of significance.

Our results show that the overall extent of adaptive evolution of protein sequences among *D. melanogaster* and *D. yakuba* is higher for male-biased genes than for female-biased or unbiased genes (figure 1), with a particularly clear pattern for *ω_a_* for the autosomes (figure 1*b*). The rate of adaptive evolution is significantly higher for the X chromosome than the autosomes for each category of gene expression (table 1).

**Table 1.** Comparisons of *ω_a_* between X- and autosomal-linked genes among different categories of sex-biased gene expression. The table displays the means and 95% bootstrap confidence intervals (in parentheses) of *ω_a_* for each category of gene expression.

expression bias	X	A	Mann–Whitney *p*-value
male	0.159 (0.144, 0.174)	0.095 (0.083, 0.109)	0.0008
unbiased	0.083 (0.072, 0.091)	0.051 (0.039, 0.064)	0.014
female	0.101 (0.091, 0.112)	0.045 (0.035, 0.057)	2 × 10^−7^

**Figure 2.**
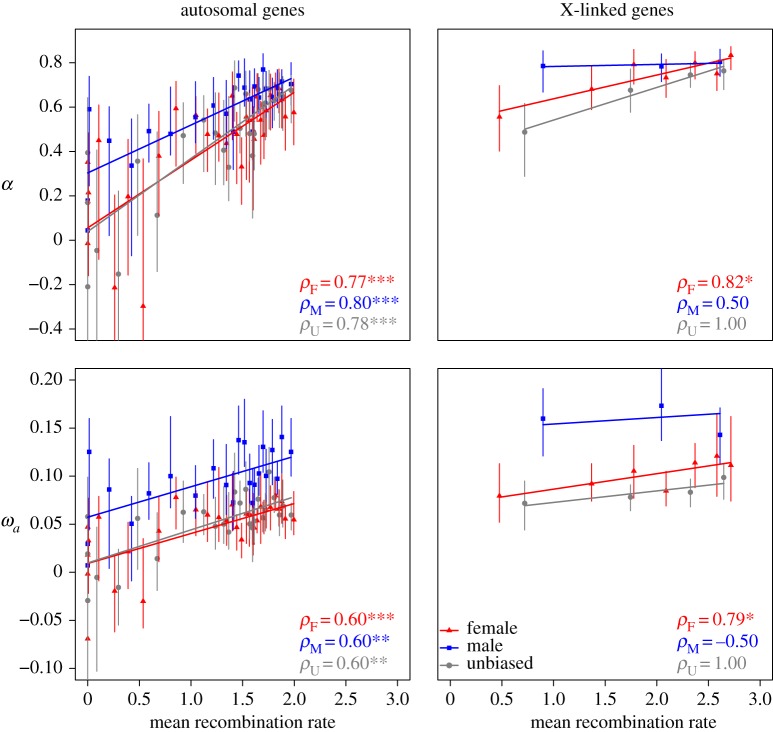
Plots of *α* and *ω_a_* against the mean effective recombination rates of bins of 80 genes (least-squares regression lines are shown), for female-biased (F), male-biased (M) and unbiased (U) genes. *ρ* denotes the Spearman's rank correlation coefficients for each expression group. * , ** and *** indicate *p* values of 0.05, 0.01 and 0.001, respectively.

These patterns might, at least in part, reflect differences in recombination rates, as higher recombination rates have previously been shown to be associated with higher *α* and *ω_a_* [5]. However, when *α* and *ω_a_* are regressed against the recombination rate for bins of 80 genes (see §2), it is evident that, while the extent of adaptive evolution is positively correlated with the recombination rate, the differences between X and autosomes and the effect of male-biased gene expression are maintained across the range of recombination rates (figure 2 and electronic supplementary material, figure S1). The frequency distributions of recombination rates are very similar for each of the three categories of gene expression, so that there is no evidence for substantial recombination rate differences among the different categories (electronic supplementary material, figures S3 and S4).

## 4. Discussion

The results described above show that the extent of adaptive Faster-X evolution in *D. melanogaster* and its relatives is not strongly affected by moderate levels of sex-biased gene expression. Although male-biased genes on the X chromosome appear to have the highest value of *ω_a_*, an analysis of variance showed no significant interaction between expression category and X versus autosome in their effects on *ω_a_* (electronic supplementary material, table S6). These results are broadly consistent with our previous findings [4]. These findings were, however, based purely on the use of *K_A_* and *K_S_*, and hence could not disentangle the contribution of adaptive substitutions to differences between X and autosomes.

Theory predicts that genes expressed only in males should be the most likely to experience adaptive Faster-X evolution and that genes expressed only in females should show no such effect [1]. But our measure of sex-biased gene expression was quite liberal, as we needed to have sufficiently large groups of genes to obtain accurate estimates of *α* and *ω_a_*. Accordingly, there may be substantial levels of gene expression in both sexes for our sex-biased genes, so that the theory is not contradicted by these results. Overall, sex-biased gene expression seems to have only a limited influence on the Faster-X effect, despite the expectation that this should be favoured by male-biased gene expression and disfavoured by female-biased gene expression [1,2]. Possible reasons for this observation were discussed in depth in [4]. Furthermore, the mean level of gene expression is similar for the X and autosomes, and the GC content at third coding positions is somewhat higher for the X [12], so that a lower level of X chromosome gene expression or GC content cannot contribute to the *Drosophila* Faster-X effect, in contrast to the situation in mammals [13].

The recombination rates for the X chromosome and autosomes shown in figure 2 were adjusted to give similar effective rates for the two types of chromosome (see §2). The results in this figure thus imply that recombination rates do not influence the Faster-X effect by reducing the extent of Hill–Robertson interference [14] among X-linked loci relative to autosomal loci, even though there is a higher overall effective rate of recombination on the X chromosome [5]. As found previously, the recombination rate is positively correlated with the extent of adaptive evolution of both X-linked and autosomal genes [5].

Our results show that a relatively modest level of male-biased gene expression has a major influence on the rate of adaptive evolution of protein sequence, regardless of chromosomal location. While faster rates of protein sequence evolution for genes with male functions have been reported many times in the literature [6], our study provides the firmest evidence to date that adaptive evolution across the genome is fastest for male-biased genes. Such a pattern has often been interpreted in terms of the evolutionary importance of sexual selection. Most *Drosophila* genes that are highly male-biased are expressed primarily in the male reproductive tissues [15], and hence are likely to affect sperm competition rather than behavioural phenotypes affecting male–male competition or attractiveness to females. In the present case, our liberal criterion for sex-biased gene expression means that both within-sex competition and female mate choice could play a role in causing a higher level of adaptive protein sequence evolution, leading to higher rates of adaptive evolution of genes with male-biased levels of expression than rates for other genes, on both the autosomes and the X chromosome.

## Supplementary Material

Avila et al Electronic Supplementary Material
