# Supplementary material for: The effects of sex-biased gene expression and X-linkage on rates of adaptive protein sequence evolution in Drosophila
Source: Biol Lett. 2015 Apr;11(4):20150117. doi: 10.1098/rsbl.2015.0117 (PMC4424624; doi:10.1098/rsbl.2015.0117)
Supplement: Avila et al Electronic Supplementary Material [file rsbl20150117supp1.doc]

**Gene classifications according to recombination rate and sex bias in expression**

1. The genes were ordered according to their effective recombination rates, and grouped into sets of ~80 genes within each category of gene expression

**Table S1 Numbers of genes within each group and numbers of groups (numbers within brackets)**

|  | **Female-biased** | **Male-biased** | **Unbiased** |
| --- | --- | --- | --- |
| **Autosomal genes** | 82-83 (33) | 84-83 (23) | 81-80 (26) |
| **X Linked genes** | 89-90 (7) | 84-85 (3) | 99-100 (4) |

2. The genes were grouped into sets with high, intermediate, and low effective recombination rates, using the criteria described in ref. (11) .

**Tables S3** Numbers of genes within each expression and recombination category for autosomal genes

| **Autosomal genes** | **Female** | ***** | **Male** |  | **Unbiased** |  | **Total** |
| --- | --- | --- | --- | --- | --- | --- | --- |
| **High** | 1341 | 3.6 | 942 | 3.5 | 1080 | 4.0 | 3363 |
| **Intermediate** | 1005 | 2.7 | 710 | 2.7 | 753 | 2.8 | 2468 |
| **Low** | 373 | 1.0 | 267 | 1.0 | 273 | 1.0 | 913 |
| Chi-squared *p*** | 0.85 |  | 0.77 |  | 0.41 |  |  |

**Table S4** Numbers of genes within each expression and recombination category for X linked genes

| **X Linked genes** | **Female** | ***** | **Male** |  | **Unbiased** |  | **Total** |
| --- | --- | --- | --- | --- | --- | --- | --- |
| **High** | 404 | 3.6 | 160 | 3.3 | 248 | 3.0 | 812 |
| **Intermediate** | 111 | 1.0 | 45 | 0.9 | 66 | 0.8 | 222 |
| **Low** | 113 | 1.0 | 48 | 1.0 | 84 | 1.0 | 245 |
| Chi-sq *p*** | 0.76 |  | 0.98 |  | 0.60 |  |  |

* mean recombination rates within each group, standardised by the value for the ‘low’ group.

** The chi-squared *p* values test whether the numbers of genes in each recombination group differ from the overall distribution.

**Table S5 Mean effective recombination rates for each group in approach 1.**

| Chromosome | Sex_bias | Group | mean.rec.rate |
| --- | --- | --- | --- |
| A | Female | 1 | 0 |
| A | Female | 2 | 0 |
| A | Female | 3 | 0 |
| A | Female | 4 | 0 |
| A | Female | 5 | 0.007 |
| A | Female | 6 | 0.107 |
| A | Female | 7 | 0.261 |
| A | Female | 8 | 0.391 |
| A | Female | 9 | 0.537 |
| A | Female | 10 | 0.689 |
| A | Female | 11 | 0.854 |
| A | Female | 12 | 1.047 |
| A | Female | 13 | 1.16 |
| A | Female | 14 | 1.269 |
| A | Female | 15 | 1.345 |
| A | Female | 16 | 1.4 |
| A | Female | 17 | 1.443 |
| A | Female | 18 | 1.49 |
| A | Female | 19 | 1.536 |
| A | Female | 20 | 1.565 |
| A | Female | 21 | 1.594 |
| A | Female | 22 | 1.603 |
| A | Female | 23 | 1.612 |
| A | Female | 24 | 1.64 |
| A | Female | 25 | 1.675 |
| A | Female | 26 | 1.707 |
| A | Female | 27 | 1.728 |
| A | Female | 28 | 1.769 |
| A | Female | 29 | 1.819 |
| A | Female | 30 | 1.852 |
| A | Female | 31 | 1.88 |
| A | Female | 32 | 1.913 |
| A | Female | 33 | 1.996 |
| A | Male | 34 | 0 |
| A | Male | 35 | 0 |
| A | Male | 36 | 0.014 |
| A | Male | 37 | 0.208 |
| A | Male | 38 | 0.424 |
| A | Male | 39 | 0.592 |
| A | Male | 40 | 0.802 |
| A | Male | 41 | 1.044 |
| A | Male | 42 | 1.218 |
| A | Male | 43 | 1.342 |
| A | Male | 44 | 1.409 |
| A | Male | 45 | 1.461 |
| A | Male | 46 | 1.518 |
| A | Male | 47 | 1.568 |
| A | Male | 48 | 1.597 |
| A | Male | 49 | 1.615 |
| A | Male | 50 | 1.658 |
| A | Male | 51 | 1.698 |
| A | Male | 52 | 1.727 |
| A | Male | 53 | 1.788 |
| A | Male | 54 | 1.835 |
| A | Male | 55 | 1.879 |
| A | Male | 56 | 1.969 |
| A | Unbiased | 57 | 0 |
| A | Unbiased | 58 | 0 |
| A | Unbiased | 59 | 0 |
| A | Unbiased | 60 | 0.088 |
| A | Unbiased | 61 | 0.297 |
| A | Unbiased | 62 | 0.483 |
| A | Unbiased | 63 | 0.672 |
| A | Unbiased | 64 | 0.925 |
| A | Unbiased | 65 | 1.123 |
| A | Unbiased | 66 | 1.235 |
| A | Unbiased | 67 | 1.317 |
| A | Unbiased | 68 | 1.365 |
| A | Unbiased | 69 | 1.422 |
| A | Unbiased | 70 | 1.475 |
| A | Unbiased | 71 | 1.531 |
| A | Unbiased | 72 | 1.568 |
| A | Unbiased | 73 | 1.596 |
| A | Unbiased | 74 | 1.612 |
| A | Unbiased | 75 | 1.65 |
| A | Unbiased | 76 | 1.695 |
| A | Unbiased | 77 | 1.721 |
| A | Unbiased | 78 | 1.756 |
| A | Unbiased | 79 | 1.805 |
| A | Unbiased | 80 | 1.856 |
| A | Unbiased | 81 | 1.889 |
| A | Unbiased | 82 | 1.971 |
| X | Female | 83 | 0.476 |
| X | Female | 84 | 1.369 |
| X | Female | 85 | 1.778 |
| X | Female | 86 | 2.089 |
| X | Female | 87 | 2.37 |
| X | Female | 88 | 2.582 |
| X | Female | 89 | 2.717 |
| X | Male | 90 | 0.898 |
| X | Male | 91 | 2.045 |
| X | Male | 92 | 2.612 |
| X | Unbiased | 93 | 0.721 |
| X | Unbiased | 94 | 1.745 |
| X | Unbiased | 95 | 2.325 |
| X | Unbiased | 96 | 2.648 |

**Table S6** Two-way analysis of variance for ranked values of ω_α_. Factors are sex expression bias (male-biased, female and unbiased) and chromosomal location (autosome versus X chromsome).

|  | DF | Sum Sq | Mean Sq | F value | P value |
| --- | --- | --- | --- | --- | --- |
| Sex bias in expression | 2 | 20869 | 10434 | 26.179 | **1.09 x 10^-09^** |
| Chromosomal location | 1 | 15928 | 15928 | 39.963 | **9.74 x 10^-09^** |
| Sex bias in expression x chromosomal location | 2 | 1051 | 525 | 1.318 | 0.273 |
| Residuals | 90 | 35872 | 399 |  |  |

**Table S7** Estimates of ω*_a_* and α (means and 95% confidence intervals)for sets of genes divided into groups according to chromosomal location (X versus A), recombination rate category (high, medium and low) and pattern of gene expression

| Chr. | Expr.  Class* | Recomb.  Rate** | No.  Genes | ω*_α_* | α |
| --- | --- | --- | --- | --- | --- |
| A | F | 1.7 | 1341 | 0.57 (0.54,0.61) | 0.06 (0.06,0.07) |
| A | F | 0.8 | 1005 | 0.44 (0.22,0.49) | 0.05 (0.02,0.06) |
| A | F | 0.2 | 373 | 0.29 (0.16,0.42) | 0.04 (0.02, 0.06) |
| A | M | 1.7 | 942 | 0.68 (0.64,0.72) | 0.11 (0.10,0.12) |
| A | M | 0.9 | 710 | 0.50 (0.45,0.56) | 0.09 (0.08,0.11) |
| A | M | 0.4 | 267 | 0.38 (0.20,0.57) | 0.07 (0.04,0.10) |
| A | U | 1.7 | 1080 | 0.61 (0.56,0.64) | 0.07 (0.06,0.07) |
| A | U | 0.8 | 753 | 0.29 (0.21,0.44) | 0.04 (0.02, 0.06) |
| A | U | 0.3 | 273 | 0.30 (0.15,0.44) | 0.04 (0.02,0.06) |
| X | F | 2.4 | 404 | 0.78 (0.74,0.81) | 0.11 (0.09, 0.13) |
| X | F | 1.3 | 111 | 0.75 (0.66, 0.84) | 0.12 (0.09, 0.15) |
| X | F | 0.9 | 113 | 0.51 (0.36,0.65) | 0.06 (0.04,0.08) |
| X | M | 2.4 | 160 | 0.80 (0.75,0.85) | 0.16 (0.13,0.28) |
| X | M | 1.3 | 45 | 0.81 (0.68, 0.88) | 0.19 (0.13,0.24) |
| X | M | 0.7 | 48 | 0.76 (0.53,0.88) | 0.15 (0.09,0.19) |
| X | U | 2.4 | 248 | 0.74 (0.69,0.77) | 0.09 (0.08,0.10) |
| X | U | 1.3 | 66 | 0.66 (0.51,0.77) | 0.09 (0.06, 0.11) |
| X | U | 0.8 | 84 | 0.44 (0.21,0.64) | 0.07 (0.03,0.10) |

* F, M and U denote female-biased, male-biased and unbiased genes.

** The mean effective recombination rate for each category (high, medium and category).

**Relations between the effective recombination rate and measures of the extent of adaptive evolution for approach 2**

**Figure S1** α and ω*_a_* versus recombination rate class for three categories of gene expression (blue, grey and red indicate male-biased, unbiased and female-biased genes, respectively.) ρ is the Spearman rank correlation for each expression category.

**
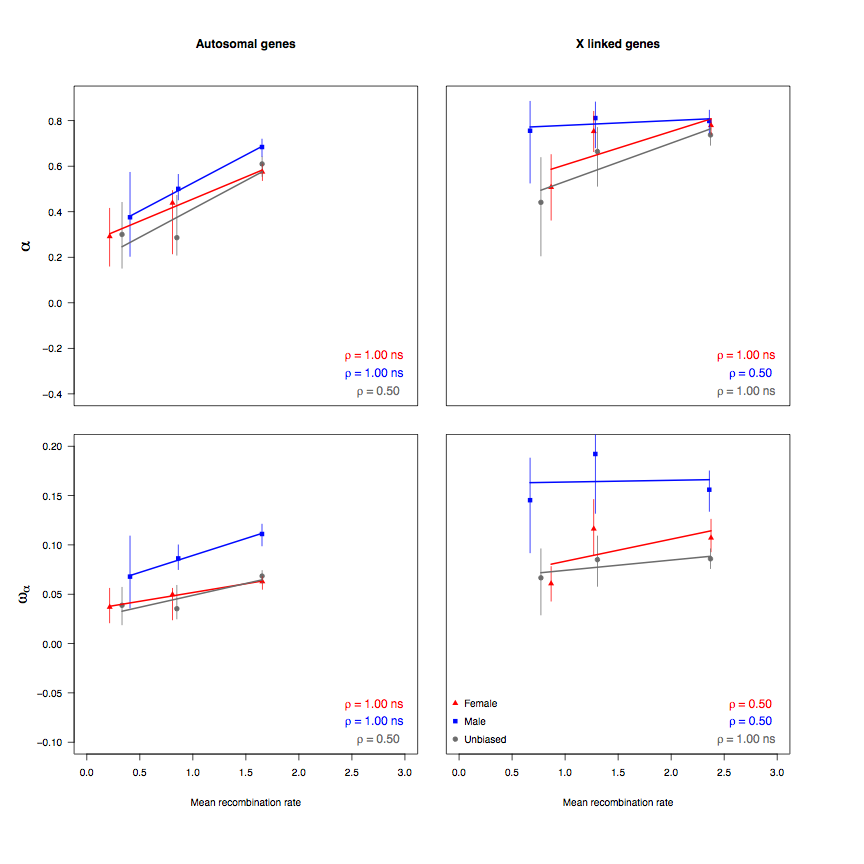
**

**Distributions of recombination rates within categories for approach 2.**

**Figure S2.** Autosomal genes

**Figure S3.** X-linked genes

These figures fail to reveal any substantial differences in the distributions of effective recombination rates among different gene expression categories.
